# Processed data on the night-time use of screen-based media devices and adolescents' sleep quality and health-related quality of life

**DOI:** 10.1016/j.dib.2019.103761

**Published:** 2019-03-07

**Authors:** Michael O. Mireku, Mary M. Barker, Julian Mutz, Chen Shen, Iroise Dumontheil, Michael S.C. Thomas, Martin Röösli, Paul Elliott, Mireille B. Toledano

**Affiliations:** a MRC-PHE Centre for Environment and Health, Department of Epidemiology and Biostatistics, School of Public Health, Imperial College London, W2 1PG, UK; b National Institute for Health Research Health Protection Research Unit in Health Impact of Environmental Hazards at King's College London, a Partnership with Public Health England, and Collaboration with Imperial College London, W2 1PG, UK; c School of Psychology, University of Lincoln, LN6 7TS, UK; d Department of Health Sciences, University of York, YO10 5DD, UK; e Social, Genetic and Developmental Psychiatry Centre, Institute of Psychiatry, Psychology and Neuroscience, King's College London, London, UK; f Department of Psychological Sciences, Birkbeck, University of London, UK; g Department of Epidemiology and Public Health, Swiss Tropical and Public Health Institute, 4051, Basel, Switzerland; h University of Basel, Switzerland

## Abstract

The data presented in this article relate to the research article entitled “Night-time screen-based media device use and adolescents' sleep and health-related quality of life”. The present data reports findings from the investigation of the relationship between night-time screen-based media devices (SBMD) use and both sleep quality and health-related quality of life (HRQoL) among 11 to 12-year-olds. Baseline data from a large cohort of 6,616 adolescents from 39 schools in and around London, UK, participating in the Study of Cognition Adolescents and Mobile Phone (SCAMP) were analysed. Self-report data on adolescents’ use of any SBMD (mobile phone, tablet, laptop, television etc.) were the main exposures of interest. Mobile phone and television were the most commonly used portable and non-portable device, respectively. Sleep variables were derived from self-reported weekday and/or weekend bedtime, sleep onset latency (SOL) and wake time. Sleep quality was assessed using four standardised dimensions from the Swiss Health Survey. HRQoL was estimated using the KIDSCREEN-10 questionnaire.

**Table:** Specifications table

Subject area	*Public Health*
More specific subject area	*Epidemiology, Psychology*
Type of data	*Tables, Figure*
How data was acquired	*Self-reported data was collected from adolescents participating in the SCAMP project using computer-based assessment and questionnaires in a classroom setting.*
Data format	*Analysed*
Experimental factors	*Data on night-time screen-based media device (SBMD) use, sleep outcomes, health-related quality of life (HRQoL) and confounding variables were obtain using questionnaires.*
Experimental features	*Sleep quality was assessed using four standardised dimensions from the Swiss Health Survey. HRQoL was assessed using the KIDSCREEN-10 questionnaire*
Data source location	*London, United Kingdom*
Data accessibility	*Summary statistics are provided in this article*
Related research article	Mireku MO, Barker MM, Mutz J, Dumontheil I, Thomas MSC, Röösli M, Elliot P, Toledano M. Night-time screen-based media device use and adolescents' sleep and health-related quality of life. Environ Int. 2019 Mar 1; 124:66–78 [1].

**Table:** 

**Value of the data** • The association between night-time SBMD use and sleep/HRQoL may be highly depending on context (e.g. geographic, social, environment). These data allow other researchers to compare their data with our cohort data. • The data informs about the relevance of different covariates in the regression modelling of night-time SBMD use and sleep/HRQoL. • SBMD use is nowadays integral part of adolescents' health and thus potentially a relevant confounder in other research areas dealing with sleep and HRQoL. Our data may help other researchers to evaluate the potential of such confounding in their study in case they have not collected such data. • The findings of the present data call for further research to understand the mechanisms underpinning the observed associations.

## 1 Data

**Fig. 1:**
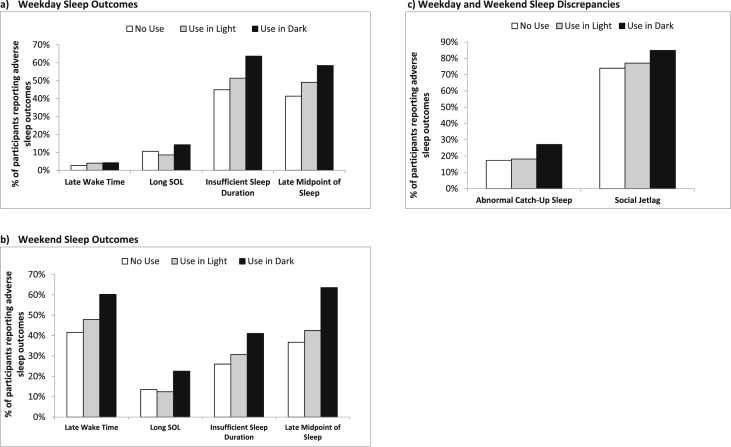
Proportion of adolescents reporting adverse sleep outcomes by night-time television watching (no use, use in light, use in darkness). Late wake time (later than 7:30 a.m. on weekdays and 8:30 a.m. on weekends); Long SOL (sleep onset latency > 45 min); Insufficient sleep duration (sleep duration <9 hr); Late midpoint of sleep (sleep midpoint later than 2:08 a.m. on weekdays and 3:53 a.m. on weekends); Abnormal catch-up sleep (difference of weekday & weekend sleep duration >2 hr); Social jetlag (difference of weekday & weekend sleep midpoint >1 hr.

The data presented in this article is complementary to the research article entitled “Night-time screen-based media device use and adolescents' sleep and health-related quality of life” [1]. In total, 52.4% of our sample were females. Females in this dataset were on average slightly younger than males (Table 1). The data investigates the association between night-time screen-based media devices (SBMD) use, implying use within 1 h before sleep, in both light and dark rooms, and sleep quality and health-related quality of life (HRQoL) among 11 to 12-year-olds. Table 2 displays the prevalence of sleep-related problems among the adolescents in the dataset. The proportion of adolescents reporting sleep-related problems on weekdays and weekends by night-time television watching (non-users, use in darkness, and use in a lit room) is shown in Fig. 1.

**Table 1:** Sociodemographic and behavioural characteristics of the 6,616 SCAMP cohort participants.

	Males (n = 3,147)	Females (n = 3,469)	*P*
Age (years), median (IQR)^a^	12.1 (11.8–12.4)	12.0 (11.8–12.3)	<0.001
BMI (kg/m^2^), median (IQR)^b^	17.5 (15.5–19.9)	17.1 (15.3–19.8)	0.235
Ethnicity			
White	1,310 (41.6)	1,359 (39.2)	0.048
Black	472 (15.0)	500 (14.4)	
Asian	745 (23.7)	925 (26.7)	
Mixed	335 (10.6)	348 (10.0)	
Other	172 (5.5)	201 (5.8)	
Missing	113 (3.6)	136 (3.9)	
Disability			
Yes	431 (13.7)	362 (10.4)	<0.001
No	2,365 (75.2)	2,696 (77.7)	
Missing	351 (11.2)	411 (11.8)	
School Type			
Independent	625 (19.9)	850 (24.5)	<0.001
State	2,522 (80.1)	2,619 (75.5)	
Parental Higher Education			
At least one	377 (11.9)	535 (15.4)	<0.001
None	1,631 (51.8)	1,723 (49.7)	
Missing	1,139 (36.2)	1,211 (34.9)	
Parental Occupation			
Higher	1,554 (49.4)	1,716 (49.5)	0.739
Intermediate	665 (21.1)	729 (21.0)	
Lower	446 (14.2)	519 (15.0)	
Missing	482 (15.3)	505 (14.6)	
Caffeine Consumption			
Yes	675 (21.4)	708 (20.4)	<0.001
No	447 (14.2)	626 (18.0)	
Missing	2,025 (64.3)	2,135 (61.5)	
Alcohol Consumption			
At least once	317 (10.1)	231 (6.7)	<0.001
Never	1,746 (55.5)	1,952 (56.3)	
Missing	1,084 (34.4)	1,286 (37.1)	
Smoking			
At least once	73 (2.3)	31 (0.9)	<0.001
Never	1,993 (63.3)	2,148 (61.9)	
Missing	1,081 (34.4)	1,290 (37.2)	
Second-hand Smoking			
Yes	608 (19.3)	693 (20.0)	0.557
No	2,349 (74.6)	2,581 (74.4)	
Missing	190 (6.0)	195 (5.6)	

BMI – Body mass index; IQR – Inter quartile range.

Unless otherwise stated, all figures are presented as number (percentage).

Missing category was not used in statistical analysis.

a *N* = 6,597.

b *N* = 1,981.

**Table 2:** Sleep quality dimensions among males and females.

	Males (n = 3,147)	Females (n = 3,469)
	n (%)	n (%)
Difficulty Falling Asleep		
Never	828 (26.3)	707 (20.4)
Rarely	1,081 (34.4)	1,126 (32.5)
Sometimes	723 (23.0)	1,003 (28.9)
Often	374 (11.9)	502 (14.5)
Missing	141 (4.5)	131 (3.8)
Sleeping Restlessly		
Never	855 (27.2)	774 (22.3)
Rarely	818 (26.0)	968 (27.9)
Sometimes	746 (23.7)	919 (26.5)
Often	587 (18.7)	677 (19.5)
Missing	141 (4.5)	131 (3.8)
Waking Up in Night		
Never	1,233 (39.2)	1,310 (37.8)
Rarely	980 (31.1)	1076 (31.0)
Sometimes	511 (16.2)	595 (17.2)
Often	282 (9.0)	357 (10.3)
Missing	141 (4.5)	131 (3.8)
Waking Up Too Early in Morning		
Never	949 (30.2)	1.093 (31.5)
Rarely	834 (26.5)	863 (24.9)
Sometimes	712 (22.6)	865 (24.9)
Often	511 (16.2)	517 (14.9)
Missing	131 (4.5)	131 (3.8)

To assess the relationship between night-time SBMD and sleep quality, we used ordered logistic regression analysis. Table 3 shows the odds of often experiencing a sleep quality problem (highest level) versus the combined lower levels of sleep quality problems (sometimes, rarely and never) among adolescents who use at least one SBMD, mobile phones or televisions at night compared to non-users.

**Table 3:** Associations between night-time use of at least one SBMD, mobile phones and televisions and sleep quality.

	SBMD	Mobile Phone	Television
	OR (95% CI)	OR (95% CI)	OR (95% CI)
Difficulty Falling Asleep			
Model I	1.56 (1.41, 1.73)^‡^	1.38 (1.26, 1.51)^‡^	1.21 (1.11, 1.33)^‡^
Model II	1.51 (1.32, 1.72)^‡^	1.39 (1.24, 1.56)^‡^	1.17 (1.04, 1.32)^#^
Model IIA	1.29 (1.04, 1.60)*	1.36 (1.11, 1.66)^#^	1.09 (0.89, 1.33)
Sleeping Restlessly			
Model I	1.61 (1.45, 1.78)^‡^	1.51 (1.38, 1.65)^‡^	1.47 (1.35, 1.61)^‡^
Model II	1.51 (1.33, 1.72)^‡^	1.39 (1.24, 1.56)^‡^	1.31 (1.17, 1.47)^‡^
Model IIA	1.37 (1.10, 1.69)^#^	1.21 (0.99, 1.48)	1.15 (0.94, 1.40)
Waking Up in Night			
Model I	1.35 (1.22, 1.50)^‡^	1.31 (1.20, 1.44)^‡^	1.38 (1.26, 1.51)^‡^
Model II	1.25 (1.09, 1.42)^#^	1.23 (1.09, 1.38)^#^	1.29 (1.15, 1.46)^‡^
Model IIA	1.00 (0.81, 1.24)	1.01 (0.83, 1.24)	1.08 (0.88, 1.32)
Waking Up Too Early in Morning			
Model I	1.28 (1.16, 1.42)^‡^	1.29 (1.18, 1.41)^‡^	1.28 (1.17, 1.40)^‡^
Model II	1.25 (1.10, 1.43)^#^	1.28 (1.14, 1.44)^‡^	1.22 (1.10, 1.34)^‡^
Model IIA	1.30 (1.05, 1.61)*	1.43 (1.17, 1.74)^#^	1.21 (0.99, 1.48)

Reference group for all models: no night-time use; *p < 0.05, #*p* < 0.01, ‡*p* < 0.001.

SBMD- Screen-based media device.

Model I: un-adjusted.

Model II: adjusted for sex, age, ethnicity, school type, parental occupation, and parental education.

Model IIA (Sensitivity analysis): Model II further adjusted for BMI, second-hand smoking, alcohol and caffeine consumption.

Table 4 shows the associations between night-time use of mobile phone or television, in darkness or in a room with the light on, and the HRQoL of adolescents. Table 4 also displays the crude or unadjusted model (Model I) and Model I adjusted for sex, age, ethnicity, school type, parental occupation, and parental education (Model II).

**Table 4:** Association between night-time mobile phone and television use (in a light/dark room) and HRQoL.

	Mobile Phone Use		Television Use	
	Light	Dark	Light	Dark
	Beta (95% CI)	Beta (95% CI)	Beta (95% CI)	Beta (95% CI)
KIDSCREEN-10 Score				
Model I	−0.43 (−0.99, 0.12)	−1.22 (−1.73, −0.70)‡	−0.04 (−0.58, 0.50)	−0.21 (−0.78, 0.36)
Model II	−0.38 (−1.06, 0.30)	−1.18 (−1.85, −0.52)^#^^a^	−0.35 (−1.02, 0.32)	0.26 (−0.50, 1.01)
Model IIA	−0.11 (−1.17, 0.96)	0.77 (−0.38, 1.92)	0.44 (−0.63, 1.50)	1.96 (0.67, 3.25)^#^
Model IIB	−0.46 (−1.19, 0.26)	−1.20 (−1.92, −0.48)^#^	−0.67 (−1.39, 0.04)	0.18 (−0.64, 0.99)

Reference group: no night-time use; #*p* < 0.01; ‡*p* < 0.001 compared to the reference group.

Model I: un-adjusted.

Model II: adjusted for sex, age, ethnicity, school type, parental occupation, and parental education Model IIA: (Sensitivity analysis): Model II further adjusted for BMI, second-hand smoking, alcohol and caffeine consumption.

Model IIB: (Sensitivity analysis): Model II excluding participants with disabilities.

a *p* < 0.05 for the comparison of the observed measure of effect between device use in darkness and in a lit room.

## 2 Experimental design, materials, and methods

### 2.1 Sample and setting

This article presents cross-sectional analysis of baseline data from the Study of Cognition, Adolescents and Mobile Phones (SCAMP) [2]. SCAMP is a prospective cohort study investigating whether children's use of mobile phones and other wireless technologies is associated with neurocognitive and behavioural outcomes [3]. The SCAMP cohort consists of 11 to 12-year-old adolescents who were recruited from 39 secondary schools in and around London, UK. For the purpose of this data, self-report information on their SBMD use and sleep and HRQoL outcomes were collected from the adolescents using a computer-based assessment in a classroom setting.

### 2.2 Exposures

The data includes adolescents’ response to questions about their use any of the following SBMD: mobile phone, tablet, eBook reader, laptop, portable media player, portable video game console, desktop computer, television or video game console, within 1 h before sleep). When adolescents affirmed their use of any of these devices, they were subsequently asked, for each type of device, if they usually use it with the light on in the room or in darkness.

### 2.3 Outcomes

#### 2.3.1 Sleep outcome measures

Adolescents reported their usual sleep patterns separately on weekdays and weekends. Specifically, they responded to questions about their bedtime, sleep onset latency (SOL), and wake time. Weekday and weekend wake times were provided as 30-min interval categories (e.g. 06:00–06:30 a.m.) anchored at “before 06:00 a.m.” and “later than 02:00 p.m.”. Similar 30-min interval categories were used for bedtimes anchored at “before 08:30 p.m.” and “later than 03:00 a.m.” for weekday nights and “before 08:00 p.m.” and “later than 03:00 a.m.” for weekend nights. From the responses provided, recommendations of the NSF [4] and the normal school start times of adolescents in London, categorical variables were created to differentiate between poor and good sleep hygiene:

(i) late weekday wake time (weekday wake time later than 7:30 a.m.);
(ii) late weekend wake time (weekend wake time later than 8:30 a.m.);
(iii) long SOL (SOL longer than 45 minutes);
(iv) insufficient sleep duration (sleep duration less than 9 hours);
(v) late midpoint of sleep (later than the sample median sleep midpoint);
(vi) abnormal catch-up sleep (weekday-weekend sleep duration difference exceeding 2 hours);
(vii) social jetlag (weekday-weekend midpoint of sleep difference exceeding 1 hour).

Sleep quality was assessed using four standardised dimensions from the Swiss Health Survey: difficulty falling asleep, sleeping restlessly, waking up several times during the night and waking up too early in the morning [5]. Adolescents were asked how often they had encountered these sleep quality problems during the last four weeks using a four-point Likert scale (Never, Rarely, Sometimes, and Often).

#### 2.3.2 Health-related quality of life measure

HRQoL was assessed using the KIDSCREEN-10, a unidimensional 10-item self-report instrument covering physical, psychological and social dimensions of wellbeing validated for use among children and adolescents aged 8 to 18-years-old [6]. For each of the 10 items, adolescents were asked to indicate the frequency or severity using a five-point Likert scale (1 = never, 2 = almost never, 3 = sometimes, 4 = almost always, and 5 = always) or (1 = not at all, 2 = slightly, 3 = moderately, 4 = very, and 5 = extremely). The total score (range: 18.5–83.8) for each participant was calculated as described elsewhere, with higher score indicating better HRQoL [6].

### 2.4 Covariates

**Fig. 2:**
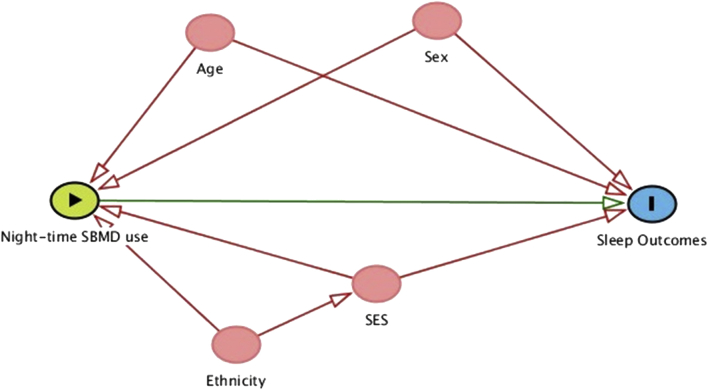
Simplified directed acyclic graph (DAG) showing selected confounders for the association between night-time use of screen-based media devices (SBMD) and sleep outcomes. Night-time SBMD use is the principal exposure and Sleep outcomes are the outcomes of interest. From a complex DAG, age, sex, socio-economic status (SES) and ethnicity were selected as potential confounders since they were common antecedents of the exposure and outcome of interest. *The same set of variables were selected as confounders when considering HRQoL as the outcome*.

Sociodemographic and behavioural characteristics of the adolescents including age, sex, weight, height, ethnicity, caffeine consumption, alcohol consumption, smoking and exposure to second-hand smoking, parental occupation and parental level of education were collected during the computer-based school assessment. Potential confounding variables were selected from the above list of covariates using directed acyclic graphs (DAGs) [7], defined as the common antecedents of exposure and outcome (see Fig. 2). With the DAG, the direction of the arrow was assumed to move from SBMD use to sleep outcomes or HRQoL. DAGs provide a structural approach to examine the relationship between an exposure and outcome to avoid adjusting for variables that introduce biases into the association [8]. Parental occupation, parental education and school type (private versus state) were used as proxy data for the socioeconomic status of the adolescent.

### 2.5 Statistical analysis

The distributions of exposure, outcome and covariate variables were checked independently and descriptive analyses were performed for these variables. Complete case analysis was employed in all statistical analyses. Two main statistical methods were used for inferential analysis:

(i) Ordered logistic regression was performed to assess the relationship between each of the SBMD exposure variables and sleep quality items.
(ii) Linear regression was used to examine the association between each of the SBMD exposure variables and KIDSCREEN-10 score.

Crude models (Model I) were run to show the unadjusted relationship between the exposures and outcomes. All models were then adjusted (Model II) for ethnicity, age, sex, school type, parental education, and parental occupation (using the National Statistics Socio-Economic Classification with 3 categories) as potential confounders based on the DAG.

As sensitivity analysis, the adjusted model was further adjusted for other covariates (body mass index [BMI], second-hand smoking, and alcohol and caffeine consumption) in Model IIA. Due to the uncertainty of the direction of the causal path between these covariates and the exposure variable i.e. potential of being on the casual pathway between the exposure and the outcome, these covariates were not included in the adjusted model (Model II). For the linear regression models with KIDSCREEN-10 score as an outcome variable, further sensitivity analyses were conducted by excluding adolescents who self-reported any disability from the analysis (Model IIB).

All analyses were conducted using Stata version IC/13.1 for Windows (StataCorp, TX). Statistical significance was defined as P < 0.05.

### 2.6 Ethical approval

The North West Haydock Research Ethics Committee approved the SCAMP protocol and subsequent amendments (ref 14/NW/0347). Head teachers of schools consented to participation in SCAMP. Parents and adolescents were provided in advance with written information and were given the opportunity to opt out of the research. The adolescents were also provided with the opportunity to opt-out of participation on the day of the assessment. The opt-out recruitment approach was expected to improve participation in an ethnically diverse population, reduce selection bias, ensure feasibility of classroom-based assessment and ensure a cost-effective study. The study was conducted in accordance with the Declaration of Helsinki.
