# Supplementary material for: Processed data on the night-time use of screen-based media devices and adolescents' sleep quality and health-related quality of life
Source: Data Brief. 2019 Mar 7;23:103761. doi: 10.1016/j.dib.2019.103761 (PMC6661065; doi:10.1016/j.dib.2019.103761)
Supplement: Multimedia component 1 [file mmc1.docx]

**Conflict of Interest Form**

We, the listed authors, wish to confirm that there are no known conflicts of interest associated with this publication and there has been no significant financial support for this work that could have influenced its outcome.

We confirm that the manuscript has been read and approved by all named authors and that there are no other persons who satisfied the criteria for authorship but are not listed. We further confirm that the order of authors listed in the manuscript has been approved by all of us.

We confirm that we have given due consideration to the protection of intellectual property associated with this work and that there are no impediments to publication, including the timing of publication, with respect to intellectual property. In so doing we confirm that we have followed the regulations of our institutions concerning intellectual property.

We further confirm that any aspect of the work covered in this manuscript that has involved either experimental animals or human patients has been conducted with the ethical approval of all relevant bodies and that such approvals are acknowledged within the manuscript.

Authors:

Dr. Michael O. Mireku

Miss Mary M. Barker

Mr. Julian Mutz

Dr. Chen Shen

Dr. Iroise Dumontheil

Prof. Michael S.C. Thomas

Prof. Martin Röösli

Prof. Paul Elliott

Prof. Mireille B. Toledano (corresponding author)
